# *Canid herpesvirus 1* Preferentially Infects Polarized Madin-Darby Canine Kidney Cells from the Basolateral Surface

**DOI:** 10.3390/v14061291

**Published:** 2022-06-14

**Authors:** Mohamed Eisa, Samar Micky, Angela Pearson

**Affiliations:** Centre Armand-Frappier Santé Biotechnologie, Institut National de la Recherche Scientifique, 531 Boul. des Prairies, Laval, QC H7V 1B7, Canada; mohamed.eisa@inrs.ca (M.E.); samar.micky@inrs.ca (S.M.)

**Keywords:** *Canid herpesvirus 1*, polarized MDCK cells, lamellipodia, scanning electron microscopy, virus-host cell interaction

## Abstract

*Canid herpesvirus 1* (CHV-1) infects polarized canine epithelia. Herein, we present our initial work characterizing CHV-1 infection of Madin-Darby canine kidney (MDCK) cells that were polarized on trans-wells. We previously showed that infection of these cells in non-polarized cultures stimulated the formation of extensive lamellipodial membrane protrusions. Uninfected polarized MDCK cells already form extensive lamellipodial membrane protrusions on the apical surface in the absence of virus. Using scanning electron microscopy, we found that CHV-1 infection does not lead to a change in the form of the lamellipodial membrane protrusions on the apical surface of polarized MDCK cells. We found that CHV-1 was able to infect polarized cultures from either the apical or basolateral side; however, higher viral titers were produced upon infection of the basolateral side. Regardless of the side infected, titers of virus were higher in the apical compartment compared to the basal compartment; however, these differences were not statistically significant. In addition to cell-free virus that was recovered in the media, the highest amount of virus produced remained cell-associated over the course of the experiment. The efficiency of CHV-1 infection of the basolateral side of polarized epithelial cells is consistent with the pathobiology of this varicellovirus.

## 1. Introduction

*Canid herpesvirus 1* (CHV-1) is a member of the *Varicellovirus* genus. The virus infects domesticated dogs as well as other canines such as foxes. CHV-1 causes a relatively mild infection in adult dogs; however, in newborn pups, the infection can be lethal. Moreover, CHV-1 causes spontaneous abortions in pregnant dams. As such, CHV-1 represents an ongoing threat to commercial breeders of domestic dogs. After the initial infection of epithelial cells by CHV-1, there is a viremia mediated by hematopoietic cells, likely T lymphocytes or monocytes. Following this systemic stage of infection, the virus infects neurons of sensory ganglia where it established latency. Upon stress or immunosuppression, the virus can reactivate leading to the production of new infectious particles in neurons. These virions ultimately infect epithelial cells near the axon termini, causing a recurrent infection [1,2]. Initially, CHV-1 binds to cells through interactions with heparan sulfate proteoglycan (HSPG) [3], likely via the viral glycoprotein C or B based on what is known from studies on other herpesviruses [4,5]. Then, entry occurs in a cell-type dependent process, likely based on specific protein–protein interactions between one or several other viral glycoproteins embedded in the viral envelope that serve as attachment proteins, and one or several cellular receptors. In the host organism, the virus initially infects epithelial cells of the respiratory or genital track [6]. We have previously shown that CHV-1 enters non-polarized Madin-Darby canine kidney (MDCK) epithelial cells by endocytosis using the cellular macropinocytic machinery [7]. Infection by the virus causes the formation of extensive lamellipodial extensions that fold back to endocytose viral particles into macropinocytic vesicles. In this report, we investigated the infection of polarized MDCK cells by CHV-1. We tested the hypothesis that CHV-1 infection causes a change in the ultrastructure of the plasma membrane extensions of polarized epithelial cells. We also investigated whether the efficiency of CHV-1 infection of MDCK cells varies according to whether the cells are infected from the apical or basolateral surface.

## 2. Materials and Methods

### 2.1. Cells and Viruses

CHV-1 strain V777 [8] was generously provided by Dr. Andrew Davison (University of Glasgow). Virus stocks were prepared and titrated by plaque assay on MDCK cells essentially as described previously [7]. MDCK cells were cultured in Dulbecco’s Modified Eagle’s Medium (DMEM) containing 8% fetal bovine serum (FBS), 50 U/mL penicillin, and 50 µg/mL of streptomycin. Cells were propagated at 37 °C in a humidified incubator with 5% CO_2_. For the plaque assays, cells were plated in 12-well plates, and the next day the confluent monolayers were infected with a serial dilution of the virus stock in duplicate, and then overlayed with DMEM containing 2% FBS and 0.4% of methylcellulose. Three days post-infection, the cells were fixed and stained with crystal violet. Plaques were visualized using a Nikon SMZ800 stereomicroscope and counted manually.

### 2.2. Polarized Cell Cultures

MDCK cells were seeded on either polyethylene terephthalate (PET) or polycarbonate transwell inserts in 6-, 12-, or 24-well plates as indicated. Each day post-seeding, the transepithelial electrical resistance (TEER) was measured as an indicator of the polarization of the culture using an EVOM2 Epithelial Volt/Ohm Meter instrument (World Precision Instruments, Sarasota, FL, USA). The TEER of the cultures reproducibly stabilized by 5 days post-seeding.

### 2.3. Confocal Microscopy

MDCK cells grown on PET inserts for 5 days in 6-well plates were processed for analysis by indirect immunofluorescence (IF). Briefly, cells were washed with PBS, and then fixed for 15 min in 4% paraformaldehyde, which was added to each compartment. Following three 5 min washes in PBS, cells were stored at 4 °C until the immunostaining step. Cells were permeabilized in 0.25% Triton X-100 (diluted in PBS) for 15 min at room temperature, then washed three times with PBS at room temperature with gentle rocking. Blocking was carried out for 1 h at room temperature using a solution of 2% BSA diluted in PBS containing 0.1% Tween 20 (PBST). Cells were then incubated with a monoclonal antibody directed against zonula occludens (ZO-1) (ZO1-1A12) (cat# 33-9100, ThermoFisher/Invitrogen, Waltham, MA, USA) at a dilution of 1:100 in the blocking buffer for 2 h, and following five 5 min washes in PBST, the cells were incubated in the dark with the secondary antibody (donkey anti-mouse coupled to Alexa Fluor 488 {cat#A-21202, ThermoFisher}) at a dilution of 1:2000. Staining for ZO-1, which is important for the formation of tight junctions in MDCK cells [9], served as a marker for polarization of the MDCK culture. After the next washing steps (PBST), nuclei were stained with 300 nM DAPI for 10 min at room temperature. Following a final round of washes (PBST), the insert fragments with the immunostained cells were mounted on slides using mowiol with the apical side of the filter towards the coverslip.

### 2.4. Scanning and Transmission Electron Microscopy

MDCK cells grown on PET inserts in 24-well plates were either left untreated, mock-infected, or infected at the indicated MOI with CHV-1 V777. Transmission electron microscopy (TEM) and scanning electron microscopy (SEM) analyses were conducted essentially as described previously [7]. At the indicated time, cells were fixed using gluteradehyde, and then processed by the INRS-Centre Armand-Frappier Santé Biotechnologie electron microscopy facility for analysis by TEM or SEM as indicated. Image acquisition for the transmission electron micrographs was carried out using a Hitachi H-7100 transmission electron microscope at 75 kV with an AMT-XRIII camera. Image acquisition for the scanning electron micrographs was carried out using a Hitachi SU-8230 instrument.

### 2.5. Viral Yield Assays

MDCK cells grown on polycarbonate inserts with 3 µm pores were mock-infected or infected with CHV-1 V777 at an MOI of 1–2. At 1 h post-infection (h.p.i.), the inoculum and the medium from each compartment were removed and replaced with 1 mL of complete medium. At 24 h.p.i., media from the apical and basolateral compartments were collected separately. Then, cells bound to the inserts were subjected to a low pH wash with 0.5 mL ice-cold citrate buffer (0.1 M citric acid, 0.1 M sodium citrate, pH3), which was added to both compartments for 30 s to remove virus associated with the external surface of the plasma membrane. Following a rinse in PBS, the membrane was excised from the insert using a razor blade and transferred to a cryotube with 1 mL of DMEM containing 8% FBS. Cells were subjected to three rounds of freeze-thawing using dry ice. The viral titer in each compartment, apical, basal, and intracellular, was then determined by standard plaque assay on MDCK cells. The final volume for each compartment was 1 mL.

## 3. Results

### 3.1. Impact of CHV-1 on Membrane Protrusions of Polarized MDCK Cells

**Figure 1:**
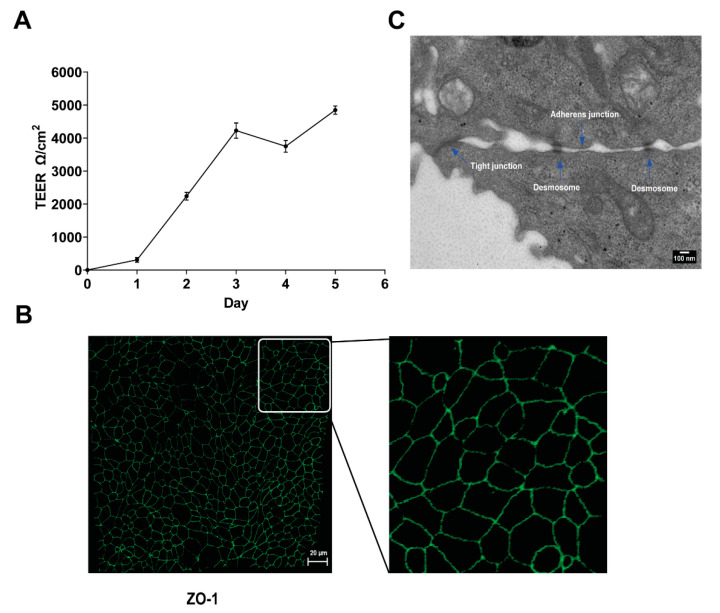
MDCK cell polarization. (**A**) Increase in value of transepithelial electrical resistance (TEER) measurements post-seeding. MDCK cells were seeded on 0.4 µm transwell cell culture inserts, and TEER was measured daily for five days after plating. Results are shown as the mean value ± SD. (**B**) Confocal image showing indirect IF staining for the tight-junction protein ZO-1 (green) expressed by polarized MDCK cells grown for five days on transwell cell culture inserts. The right-hand panel shows an enlargement of the section delineated by the white square. (**C**) Electron micrograph showing the junctional complex (tight junction, adherens junction, and desmosome) formed between polarized MDCK cells at five days post-seeding.

Because CHV-1 infection induces the formation of membrane protrusions on MDCK cells grown in monolayers in cell culture plates [7], we investigated whether CHV-1 affected the ultrastructure of the plasma membrane of polarized MDCK cells. Cultures of polarized MDCK cells were established essentially as described previously [10,11]. Transepithelial electrical resistance (TEER) was measured daily and found to rise initially and ultimately stabilize at five days post-seeding (Figure 1A). To monitor the formation of tight junctions in the polarized cultures, ZO-1 was visualized by indirect immunofluorescence using confocal microscopy. We detected strong staining at the junctions between cells at five days post-seeding (Figure 1B), thus confirming polarization of the culture. Visualization of the polarized cells by TEM revealed the formation of junctional complexes between adjacent cells, which further confirmed the polarization of the MDCK culture under our conditions (Figure 1C).

**Figure 2:**
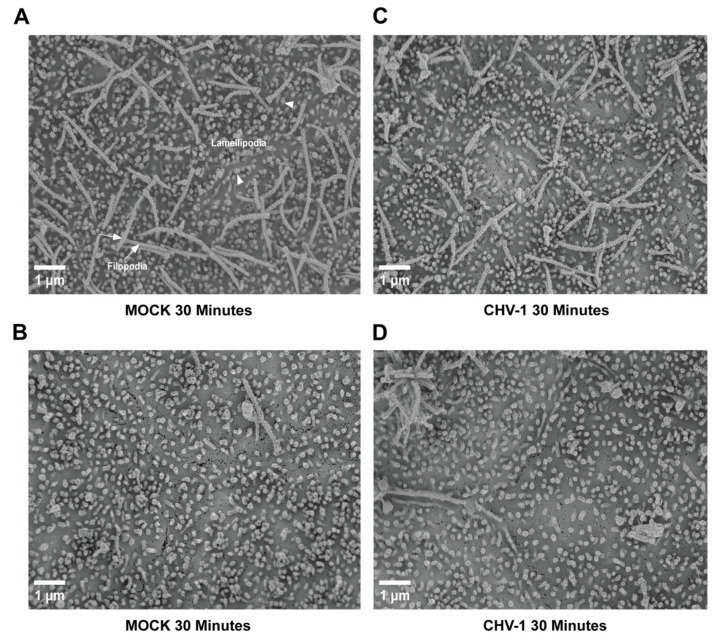
Ultrastructure of the apical surface of polarized MDCK cells is not altered upon CHV-1 entry. Electron micrographs showing the apical surface of MDCK cells polarized on transwell inserts that have either been mock-infected for 30 min (panels (**A**,**B**)) or infected with CHV-1 for 30 min (panels (**C**,**D**)). For each condition, two different sections of the cell surface are shown to illustrate the heterogeneity across the surface of the same cell. Magnification was 10,000×. Arrowheads point to examples of lamellipodia. Arrows point to examples of filopodia. Results shown are representative images from one of two independent experiments.

**Figure 3:**
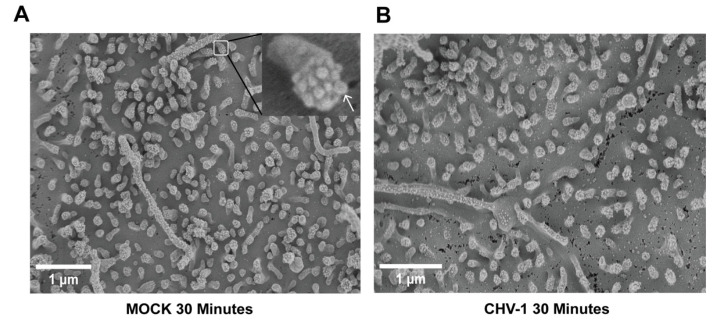
Ultrastructure of lamellipodia at the apical surface of polarized MDCK cells is not altered upon CHV-1 entry. Electron micrographs showing MDCK cells polarized on transwell inserts that have either been mock-infected for 30 min (**A**) or infected with CHV-1 for 30 min (**B**). Magnification was 20,000×. In (**A**), a section of the image is enlarged to show a single lamellipodium; an arrow points to one of the protrusions at the tip of the lamellipodium. Results shown are representative images from one of two independent experiments.

We have previously shown that CHV-1 enters non-polarized MDCK cells by a macropinocytosis-like mechanism [7]. In non-polarized cell cultures, CHV-1 infection induces the formation of extensive lamellipodial membrane ruffles, which fold back to endocytose virions. However, polarized MDCK cells exhibit extensive lamellipodial extensions even in the absence of virus [12]. Thus, we wanted to test whether CHV-1 infection of polarized MDCK cells would alter these membrane extensions. MDCK cells were seeded on PET inserts with 0.4 µm pores in 24-well cell culture plates to allow the polarization of the cells. At five days post-seeding, the cells were either mock-infected, or infected from the apical side at an MOI of 2.5. Two independent experiments were done, each of which was carried out in duplicate (i.e., two separate polarized cultures on inserts for each condition). At t = 30 min for both the mock- and CHV-1-infected conditions, the cells on the inserts were processed for SEM. Representative electron micrographs are shown in Figure 2 and Figure 3. We did not discern an obvious difference in the form of membrane ruffles of the mock-infected cells (Figure 2A,B) compared to the cells infected with CHV-1 (Figure 2C,D). For both mock-infected and CHV-1-infected cells, lamellipodial extensions were observed across the apical surface of the cell. However, the apical surface was not homogeneous, and we also observed patches where clusters of long filopodia had formed (Figure 2A,C). Similar to what we saw for the lamellipodia, the long filopodial structures were readily observed in both mock- and CHV-1-infected cells.

At higher magnification (20,000×), it was possible to visualize and count the small protrusions at the tip of the lamellipodial extensions (Figure 3). We analyzed 25 lamellipodia for each condition. No statistically significant difference was observed. The average number of these protrusions visible per lamellipodium in the context of mock-infected cells was 9.4 ± 1.5 (Figure 3A) and for CHV-1-infected cells (Figure 3B) it was 8.5 ± 1.4. We presume these values are underestimates since the angle at which an individual lamellipodium is imaged can affect the visibility of the end protrusions. In conclusion, we did not observe any differences in the apical plasma membrane surface comparing mock-infected and CHV-1-infected cells.

### 3.2. CHV-1 Infects the Basolateral Side of Polarized MDCK Cells More Efficiently than the Apical Side

**Figure 4:**
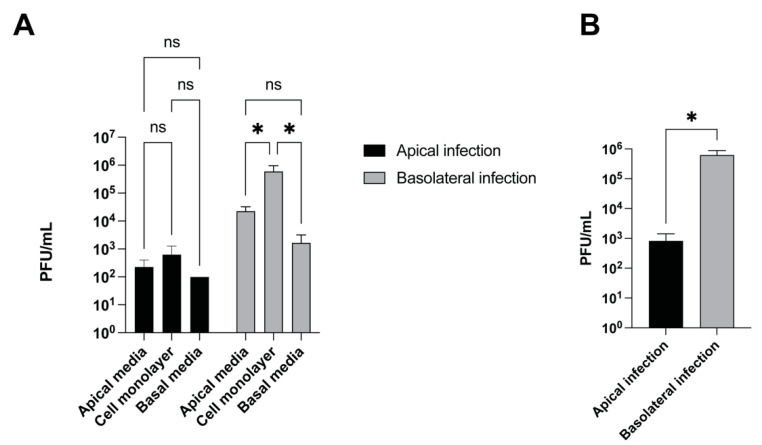
CHV-1 infection is more efficient after basolateral surface inoculation than after apical inoculation. Polarized MDCK cells grown in 12-well plates with trans-well inserts were infected either apically or basolaterally with CHV-1 V777. Virus yield at 24 h.p.i was quantified by viral plaque assay. (**A**) Virus yield for cell culture supernatants in the apical chamber, in the basolateral chamber, and from the cell monolayer were determined. Values represent the averages from two independent experiments each done in duplicate. Error bars show the standard error of the mean. Two-way ANOVA test: * *p* < 0.05. (**B**) Total virus yield from the three compartments (apical chamber, cell monolayer, basolateral chamber) for apically- and basolaterally-infected polarized MDCK cells. The results represent the averages of two independent experiments each done in duplicate (*n* = 2). Two-tailed Student’s *t*-test: * *p* < 0.05, ns, not significant.

Certain viruses differ in the efficiency with which they infect the apical or basolateral surfaces of polarized cells. For example, HSV-1 infects polarized human uterine (ECC-1), colonic (CaCo-2), and retinal pigment (ARPE-19) epithelial cells more efficiently from the apical surface [13]. Moreover, it has been reported that the asymmetric distribution of cellular receptors on polarized cells for certain viruses promotes the preferential entry of the virus from the apical or basolateral surface including Ebola virus [14] and bovine viral diarrhea virus [15]. We next tested whether CHV-1 infected the apical or basolateral surface of polarized MDCK cells more efficiently or whether there was no difference. Polarized cultures were infected with CHV-1 at an MOI of 1–2 for 1 h from either the apical or basolateral compartment, then the inoculum was removed, and the cells washed 2× with PBS and then 1× with complete DMEM media. The infected cells were then incubated for another 23 h. At 24 h.p.i., media from the apical and basal compartments were carefully collected. The cells on the insert were rinsed with low pH buffer to inactivate surface-bound virus, then collected and lysed in a volume of 1 mL, and intracellular virus was collected in the form of cell lysate. Virus from all three compartments were titrated on MDCK cells by plaque assay (Figure 4). We found that CHV-1 was able to infect MDCK cells from both the apical and basolateral surfaces (Figure 4A) leading to the production of new infectious particles.

In cells infected from the basolateral surface, we found that the amount of cell-associated virus was significantly higher than the amount of cell-free virus detected in either the apical or basolateral compartments. Although viral titers were higher in the apical compartment, the difference as compared to the amount of virus in the basal compartment was not statistically significant. Similar trends regarding the amount of virus present in the different compartments were observed for cells infected apically; however, none of these differences were statistically significant. When we compared the total amount of virus produced from a basolateral infection versus an apical infection done in parallel, we found that the basolateral infection resulted in approximately 750-fold more virus produced than the apical infection (Figure 4B).

## 4. Discussion

CHV-1 infects polarized epithelia of its host organism. As such, models of infection using polarized cells are critical to achieve a comprehensive understanding of the interaction of CHV-1 with its host. In this report, we describe our initial studies to determine how CHV-1 interacts with polarized epithelial cells, using the model of MDCK cells polarized on cell culture well porous inserts. Based on our previous results demonstrating that CHV-1 induces extensive lamellipodia formation when infecting non-polarized MDCK cells [7], we tested the hypothesis that CHV-1 would induce changes to the plasma membrane of polarized MDCK cells as well. Our SEM images showed that the ultrastructure of the apical surface of the plasma membrane of polarized MDCK cells exhibits extensive lamellipodial extensions as well as randomly distributed clusters of filopodia. CHV-1 infection did not result in broad changes to the apical plasma membrane surface, despite using the same MOI that was sufficient to induce generalized ruffling of the plasma membrane of non-polarized MDCK cells [7]. However, given the low viral titers obtained from infection of the apical surface, we cannot rule out that the effective MOI for these experiments was lower than that used previously for the non-polarized cells. At higher magnification, we were able to discern the small protrusions at the end of individual lamellipodia. However, here too, we did not detect any difference in morphology when comparing the mock-infected and CHV-1-infected cells. These results suggest that the nature of the apical surface of the polarized cells itself, which is characterized by multiple plasma membrane extensions, provides an adequate surface area to support efficient binding of the virus, and ultimately infection. However, our results do not exclude the possibility of localized alterations in the ultrastructure of the cell membrane that are restricted to sites of contact with the virus. Such localized changes have been observed in the case of infection of polarized MDCK cells by the bacterial pathogen enteropathogenic *E. coli* using corelative light and electron microscopy [16]. If so, then infection with a higher MOI than that used in these experiments might be necessary in order to easily detect such localized changes. Of note, the pathophysiology of CHV-1 infections is such that the virus not only infects epithelial cells during the initial exposure of the virus to the mucosa; during the subsequent viremia, infection of epithelial cells would be expected to occur from the basolateral side. Moreover, during viral reactivation from latency in the neurons of sensory ganglia, the newly formed virus is released from the ends of the axons and goes on to infect epithelia [1], once again presumably from the basolateral side, causing recurrent lesions and promoting spread of the virus to new hosts. Thus, efficient infection of epithelial cells from the basolateral side is an important step to ensure efficient spread of the virus within a population. The importance of this step may explain our results showing that CHV-1 infection is more efficient from the basolateral surface than from the apical surface of polarized MDCK cells. However, the mechanisms responsible for this difference remain to be determined. One possibility may be an asymmetric distribution of cellular receptors for the virus, with preferential expression on the basolateral surface. It has been shown that asymmetric distribution of nectin-1, one of the HSV-1 receptors, contributes to the preferential infection of the apical surface of polarized epithelial cells by this virus [13]. Furthermore, because we found that CHV-1 can infect both the apical and basolateral surfaces of MDCK cells, we cannot rule out the possibility that different receptors mediate entry at each of the two surfaces, which appears to be the case for infection of bovine airway epithelial cells by bovine diarrhea virus [15]. Identification of the cellular receptor(s) for CHV-1 will be an important step towards answering this question. Moreover, regardless of whether the receptors are the same, it is possible that different entry pathways for the virus are utilized at the apical and basolateral surfaces, which could differ with respect to their efficiency for virus entry. It is also possible that the difference in efficiency of CHV-1 infection from the two sides of polarized epithelial cells is due to a post-entry event such that once the virion is internalized, the membrane fusion and subsequent trafficking mechanisms that bring the capsid to the nucleus are more efficient when the virus enters at the basolateral surface. Live cell imaging experiments exploiting fluorescently labeled virus could be useful to study post-entry dynamics of the virus. The use of novel complex model systems of CHV-1 infection of epithelial cells, such as organ culture models [17], will be useful for understanding the mechanisms of CHV-1 infection of polarized epithelia in host organisms. Ultimately, a better understanding of the interaction of CHV-1 and polarized epithelial cells will help identify targets for the development of novel treatments to inhibit the initial infection as well as to limit transmission to new hosts.

## Data Availability

Data are contained within the article.
